# Effect of Lipid Head Groups on Double-Layered Two-Dimensional Crystals Formed by Aquaporin-0

**DOI:** 10.1371/journal.pone.0117371

**Published:** 2015-01-30

**Authors:** Richard Kevin Hite, Po-Lin Chiu, Jan Michael Schuller, Thomas Walz

**Affiliations:** 1 Department of Cell Biology, Harvard Medical School, Boston, Massachusetts, United States of America; 2 Howard Hughes Medical Institute, Harvard Medical School, Boston, Massachusetts, United States of America; University of Bern, SWITZERLAND

## Abstract

Aquaporin-0 (AQP0) is a lens-specific water channel that also forms membrane junctions. Reconstitution of AQP0 with dimyristoyl phosphatidylcholine (DMPC) and *E. coli* polar lipids (EPL) yielded well-ordered, double-layered two-dimensional (2D) crystals that allowed electron crystallographic structure determination of the AQP0-mediated membrane junction. The interacting tetramers in the two crystalline layers are exactly in register, resulting in crystals with *p*422 symmetry. The high-resolution density maps also allowed modeling of the annular lipids surrounding the tetramers. Comparison of the DMPC and EPL bilayers suggested that the lipid head groups do not play an important role in the interaction of annular lipids with AQP0. We now reconstituted AQP0 with the anionic lipid dimyristoyl phosphatidylglycerol (DMPG), which yielded a mixture of 2D crystals with different symmetries. The different crystal symmetries result from shifts between the two crystalline layers, suggesting that the negatively charged PG head group destabilizes the interaction between the extracellular AQP0 surfaces. Reconstitution of AQP0 with dimyristoyl phosphatidylserine (DMPS), another anionic lipid, yielded crystals that had the usual *p*422 symmetry, but the crystals showed a pH-dependent tendency to stack through their cytoplasmic surfaces. Finally, AQP0 failed to reconstitute into membranes that were composed of more than 40% dimyristoyl phosphatidic acid (DMPA). Hence, although DMPG, DMPS, and DMPA are all negatively charged lipids, they have very different effects on AQP0 2D crystals, illustrating the importance of the specific lipid head group chemistry beyond its mere charge.

## Introduction

The lens-specific water channel aquaporin-0 (AQP0) forms large square arrays in the membranes of fiber cells that allow formation of cell-cell junctions between adjacent cells [1,2]. Reconstitution of purified AQP0 with lipids resulted in double-layered, two-dimensional (2D) crystals that recapitulated the *in vivo* junctions [3,4]. Electron crystallography of these 2D crystals revealed the structure of AQP0 at 1.9 Å resolution, as well as a nearly complete model of the lipid bilayer surrounding the channel; the lipids were modeled as dimyristoyl phosphatidylcholine (DMPC), the synthetic lipid used for 2D crystallization (AQP0_DMPC_) [5]. As DMPC is not present in native lens membranes, the interactions formed between AQP0 and DMPC were representative of the non-specific interactions that all membrane proteins form with their annular shell of lipids.

To characterize the nature of non-specific lipid—protein interactions, AQP0 2D crystals were grown with *E*. *coli* polar lipids (EPL) and analyzed by electron crystallography [6]. Despite differences between DMPC and EPL in both their head group chemistry and the length and saturation state of their acyl chains, the overall organization of the lipid bilayers was quite similar in the two structures. In particular, the positions of the acyl chains are nearly identical, especially in the extracellular leaflet, providing evidence that annular lipids occupy preferred positions on the surface of membrane proteins. However, the positions of the polar head groups varied between the two structures, and the head groups of corresponding lipid molecules interacted with different protein residues, suggesting the positions of the annular lipids are defined by hydrophobic van der Waals interactions between the lipid acyl chains and the protein rather than by electrostatic interactions between the polar lipid head groups and the protein.

Notably, phosphatidylethanolamine (PE), the predominant head group found in EPL, and phosphatidylcholine (PC) are both zwitterionic, which may explain the lack of interactions between AQP0 and the lipid head groups in the structures, as many of the specific lipid—protein interactions identified in biochemical assays or resolved in crystal structures occur between membrane proteins and anionic phospholipids [7]. To test whether head groups of anionic lipids would form specific interactions with AQP0, we grew 2D crystals of AQP0 with phosphatidyl glyercerol (PG), phosphatidyl serine (PS) and phosphatidic acid (PA) lipids, lipids with three different anionic head groups. In the course of these reconstitution experiments, we made the unexpected observation that each of the anionic lipids had a unique effect on the AQP0 2D crystals that formed.

## Materials and Methods

### Protein purification and 2D crystallization

The core tissue of sheep lenses (purchased from Wolverine Packing Company, Detroit, MI) was dissected away from the soft cortical tissue and used to prepare membranes as described previously [4]. Membranes were solubilized in 4% (w/v) octyl glucoside (OG) in 10 mM Tris, pH 8.0, for 30 minutes at room temperatures. Insoluble material was removed by centrifugation at 300,000×g for 60 minutes at 4°C. Solubilized proteins were bound to a MonoQ column (GE Healthcare) and eluted with 150 mM NaCl in 1.2% (w/v) OG in 10 mM Tris, pH 8.0. Peak fractions were pooled and run over a Superose 12 column (GE Healthcare) in 1.2% OG, 10 mM Tris, pH 8.0, 100 mM NaCl. Purified AQP0 was reconstituted into 2D crystals with different lipids (DMPG, DMPS, and DMPA) or lipid mixtures (DMPG/DMPE and DMPA/DMPE) at a lipid-to-protein ratio (LPR) of 0.6 (w/w) by dialysis in 50-μl dialysis buttons (Hampton Research) against 2 liters of 10 mM MES, pH 6.0, 100 mM NaCl, 50 mM MgCl_2_, 0.05% (w/v) NaN_3_ at 37°C for one week with daily buffer exchanges. The dialysis buffers used for reconstitutions at different pH were 10 mM citrate for pH 4.0, 10 mM MES for pH 6.0, 10 mM Tris for pH 8.0, and 10 mM glycine/NaOH for pH 10.0. For dialysis of pre-formed 2D crystals, the crystal solution was dialyzed against buffers of the desired pH at 37°C overnight.

### Data collection

Specimens for cryo-EM were prepared using the carbon sandwich technique [8]. An additional step was introduced, in which the grid was blotted from the top before applying the second carbon film. This blotting step ensured that a proper thickness of the trehalose layer was obtained during specimen preparation, which was essential for obtaining good diffraction patterns [9]. Grids were transferred into a Polara electron microscope (FEI) operated at an acceleration voltage of 300 kV. Diffraction patterns were recorded with a 4K×4K Ultrascan CCD camera (Gatan) and a camera length of 1.9 m. Images were also taken with the 4K×4K CCD camera using a nominal magnification of 80,000× and a defocus setting of-400 to-1500 nm.

### Image processing

Images of 2D crystals were computationally unbent and corrected for the effects of the contrast transfer function using the 2dx software [10]. Projection maps of untilted crystals formed with DMPG were initially calculated without applying symmetry operators. Only after visual inspection of the map and analyzing the symmetry by ALLSPACE [11] was the appropriate symmetry applied. The images of untilted 2D crystals of AQP0 grown with all other lipids or lipid mixtures were analyzed using the same method; all crystals showed *p*422 symmetry, which was applied to the projection maps.

For 3D merging of AQP0_DMPG_, images of untilted crystals were classified by their plane group using ALLSPACE [11] and merged in 2dx [12]. Images of tilted crystals were merged using the previously classified and merged untilted images as references in 2dx. Merging was repeated in an iterative fashion until all of the images were classified into three groups: *p*42_1_2, *p*12_1_, and other crystal forms. Density maps for the merged images of the *p*42_1_2 and *p*12_1_ crystals were calculated at 8 Å and 7 Å, respectively, after applying a negative temperature factor of 500 to boost the high-resolution information. The statistics for the 3D density maps are presented in Table 1, and the maps were submitted to the EMDatabank with accession numbers EMD-5802 (AQP0_DMPG-p4212_) and EMD-5803 (AQP0_DMPG-p121_).

**Table 1:** Electron crystallographic statistics for the 3D reconstructions obtained with AQP0 2D crystals grown with DMPG with *p*42_1_2 symmetry (AQP0_DMPG-p4212_) and *p*12_1_ symmetry (AQP0_DMPG-p121_).

	AQP0_DMPG-*p*4212_	AQP0_DMPG-*p*121_
Crystal parameters		
Layer group	p4212	p121
Cell dimensions	a = b = 65.5 Å, γ = 90.0°	a = b = 65.5 Å, γ = 90.0°
Assumed thickness	200 Å	200 Å
Merging		
Number of images merged	227 (0°: 5; 20°: 24; 45°: 124; 60°: 73)	146 (0°: 34; 20°: 29; 45°: 64; 60°: 17)
Resolution limit for merging	7 Å	8 Å
Number of amplitudes and phases	19,860	15,374
Unique reflections	1,149	2,826
Overall weighted phase residual	16.8°	27.8°
Overall weighted R-factor	32.2%	30.0%
Completeness	84.2%	62.4%

### Docking

UCSF Chimera [13] was used to manually dock the atomic model of AQP0_DMPC_ (PDB ID: 2B6O) [5] into the low-resolution density maps, and the CCP4 program suite was used for non-crystallographic symmetry averaging and rigid body refinement [14]. Figures were generated with PyMOL (The PyMOL Molecular Graphics System, Schrodinger, LLC) and UCSF Chimera.

## Results and Discussion

### DMPG causes AQP0 to form 2D crystals with different symmetries

We first reconstituted AQP0 with dimyristoyl phosphatidylglycerol (DMPG), an anionic phospholipid whose head group is composed of a negatively charged phosphodiester group and an uncharged glycerol moiety (S1A Fig.). Since DMPC, the lipid used to form the first AQP0 2D crystals [4], and DMPG differ exclusively in their head groups, any observed differences in lipid—protein interactions can be directly attributed to the difference in head group chemistry.

**Fig 1:**
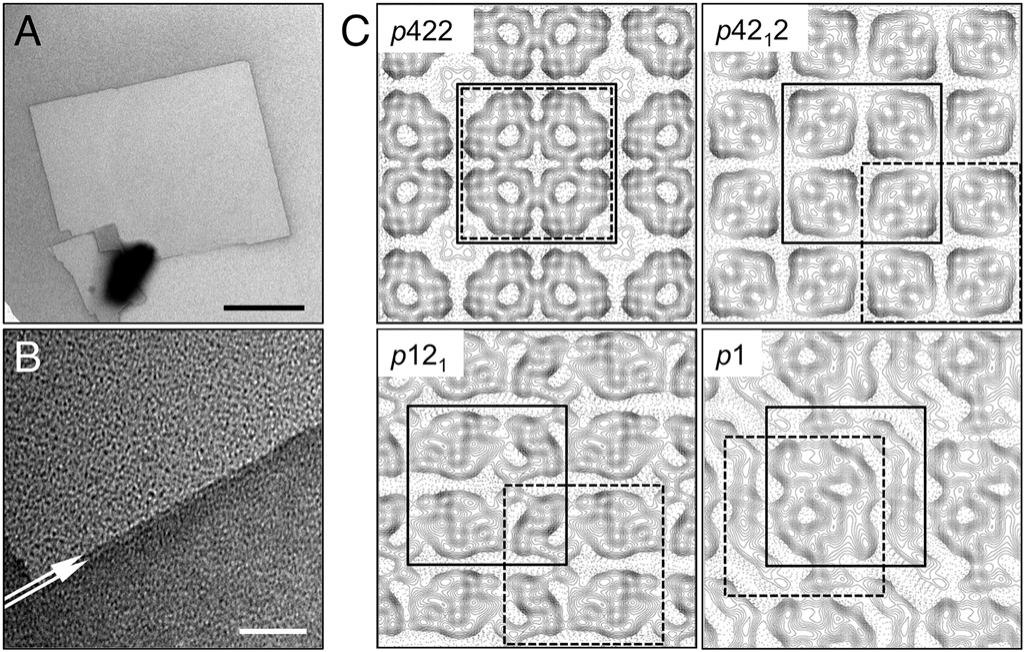
Double-Layered 2D Crystals of AQP0 Grown with DMPG. **A** Image of a representative AQP0 2D crystal formed with DMPG in negative stain. Scale bar is 1 μm. **B** High magnification view of an edge of the crystal. The white arrows mark the two parallel edges of the double-layered crystal. Scale bar is 50 nm. **C** Contour representations of four projection maps at ~7 Å resolution calculated from images of AQP0 2D crystals with *p*422, *p*42_1_2, *p*12_1_, and *p*1 symmetry (see also S1 Table). All crystal forms have the same unit cell of *a* = 65.5 Å, *b* = 65.5 Å, and γ = 90.0°. The solid and dashed squares indicate the relative position of AQP0 tetramers in the two crystalline layers of the double-layered crystals. For projection maps other than that of the crystal with *p*422 symmetry, the locations of the tetramers were determined by cross correlation with the *p*422 projection map. The different crystal symmetries result from shifts between the two crystalline layers in the direction parallel to the lattice vectors.

AQP0 was reconstituted with DMPG as described before [6], yielding large 2D crystals (Fig. 1A) that produced electron diffraction patterns with strong reflections beyond a resolution of 2 Å (S1B, C Fig.). We processed more than 600 diffraction patterns of 2D crystals collected at various tilt angles. Although the crystals were double-layered, as seen by the two parallel edges of crystals prepared in negative stain (Fig. 1B), and had unit cell dimensions identical to those of AQP0 2D crystals grown with DMPC and EPL, *a* = *b* = 65.5 Å, and γ = 90°, we were not able to merge the diffraction patterns using the established *p*422 symmetry of double-layered AQP0 2D crystals [4,6]. When we collected low-dose images of the crystals and used them to calculate projection maps, it became evident that instead of all crystals displaying the expected *p*422 symmetry, reconstitution of AQP0 with DMPG resulted in a mixture of double-layered 2D crystals with *p*1, *p*12_1_, *p*42_1_2, and *p*422 plane symmetries (Fig. 1C and S1 Table). Comparison of the projection map of the DMPG crystals with *p*422 symmetry with those of AQP0 in DMPC or EPL revealed no differences in the projections of AQP0 at a resolution of 7 Å, suggesting that DMPG had not changed the overall structure of the AQP0 tetramer, but rather its packing in the crystals.

Despite clear differences in the projection maps calculated from the *p*1, *p*12_1_ and *p*42_1_2 crystal forms compared with that of the *p*422 crystal form, the unit cell dimensions of all crystal forms were identical, indicating that the novel symmetries were a result of changes in the register between the two crystalline layers. Furthermore, the presence of a 2_1_ screw axis in crystals with *p*12_1_ and *p*42_1_2 symmetries is consistent with double-layered crystals, in which the two crystalline layers have opposite orientations, identical to the previously observed *p*422 crystals, adding support to the notion that the novel crystal forms resulted from lateral shifts between the two crystalline layers.

It is not uncommon for membrane proteins to form multiple 2D crystal forms when different crystallization conditions are used, as exemplified by the bacterial porin OmpF [15]. Usually, however, a given reconstitution condition yields a single crystal form. It is more rare that a given reconstitution condition yields a mixed crystal population, as for example in the case of microsomal glutathione S-transferase 1 (mGST-1) [16] and AQP4 [17]. While mGST-1 formed two major crystal forms that were very distinct both in unit cell dimensions and symmetry [16], the different double-layered 2D crystals formed by AQP4 had the same unit cell dimensions [17], as we now observe for our AQP0 crystals formed with DMPG. Thus, the differences between our AQP0 crystal forms, as for the AQP4 crystals, are due to different arrangements of the two crystalline layers, but do not result from differences within the individual crystalline layers.

### DMPG alters the interactions between the layers of double-layered AQP0 2D crystals

In order to characterize the different crystal forms and their packing interactions, we collected 700 images at tilt angles ranging from 0° to 70°. The images of the untilted crystals were first classified according to their symmetry based on the output of the program ALLSPACE [11]. Crystals with *p*12_1_ and *p*42_1_2 symmetry were most abundant in this data set, 33% and 48%, respectively, and were therefore chosen for further analysis. Images of untilted crystals in these two classes were separately merged in two dimensions using the 2dx software [10,12]. All images of tilted crystals were then merged with the *p*42_1_2 projection map of the untilted crystal, and the images with the highest phase residuals (poorest fit with the *p*42_1_2 projection map) were removed. The merging was iteratively refined, and the images with the highest phase residuals were removed after each refinement cycle, resulting in a final set of 227 images of tilted crystals representing exclusively AQP0 crystals with *p*42_1_2 symmetry. All remaining images of tilted crystals were then merged with the *p*12_1_ projection map, and the data set was again iteratively refined by removing the images with the highest phase residuals after each refinement cycle as described above, yielding a data set of 146 images of tilted crystals representing exclusively AQP0 crystals with *p*12_1_ symmetry. The reason we first tested all images of tilted crystals against *p*42_1_2 symmetry is that the higher symmetry places more constraints on the phases, resulting in more rapid convergence during merging.

**Fig 2:**
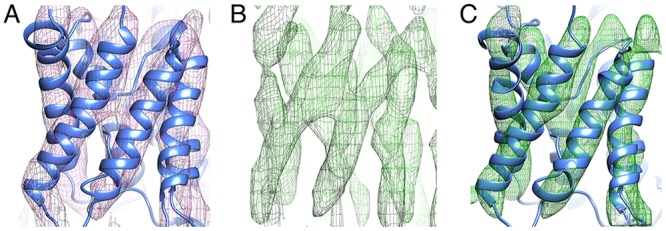
Docking of the Atomic Model of AQP0 into the 3D Density Maps of the DMPG Crystals with *p*42_1_2 and *p*12_1_ Symmetry. **A** Density map at 7 Å resolution calculated from images of AQP0 2D crystals with *p*42_1_2 symmetry (purple) with the docked atomic model (blue). **B** Density map at 8 Å resolution calculated from images of AQP0 2D crystals with *p*12_1_ symmetry before non-crystallographic symmetry (NCS) averaging. **C** The map calculated from images of AQP0 2D crystals with *p*12_1_ symmetry was improved by NCS averaging (green). The docked atomic model of AQP0 is shown in blue.

**Fig 3:**
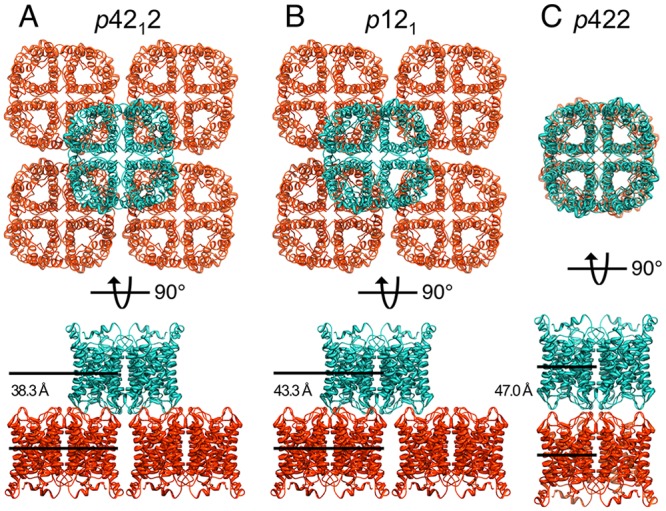
Organization of AQP0 Tetramers in the Different 2D Crystal Forms. **A** In 2D crystals formed with DMPG that show *p*42_1_2 symmetry, a tetramer in one membrane sits in the center of four tetramers in the opposing membrane, thus making contact with subunits of four separate tetramers. **B** In crystals with *p*12_1_ symmetry, the tetramer in one membrane is shifted by half a unit cell length (32.75 Å) in one lattice direction but by less than half a unit cell length in the other lattice direction (28.7 Å). As in the *p*42_1_2 crystals, each tetramer makes contact with subunits of four separate tetramers in the opposing membrane. **C** AQP0 tetramers in the two membranes in 2D crystals formed with DMPC are exactly in register, establishing *p*422 symmetry, and each tetramer thus only interacts with subunits of a single tetramer in the opposing membrane. 2D crystals formed with DMPG that show *p*422 symmetry likely have the same tetramer arrangement as the one shown here for 2D crystals formed with DMPC. The lower panels show the distances between the two layers in the different crystal forms.

Density maps were calculated from the merged image data sets to provide views of AQP0 in the *p*42_1_2 and *p*12_1_ crystal forms at 7 Å and 8 Å resolution, respectively (Table 1, S2 Fig.). The densities for the transmembrane helices were clearly resolved in the map calculated from images of the *p*42_1_2 crystal form, allowing manual docking of the atomic model of AQP0_DMPC_ (PDB ID: 2B6O) [5] (Fig. 2A). The features were less clear in the map calculated from images of the *p*12_1_ crystal form (Fig. 2B), but were noticeably improved by applying four-fold non-crystallographic symmetry averaging, which allowed unambiguous manual docking of the AQP0_DMPC_ structure (Fig. 2C). The fit of both docked models were improved by rigid-body refinement, yielding pseudo-atomic models of AQP0 in the *p*42_1_2 crystals formed with DMPG (AQP0_DMPG-p4212_) (Fig. 3A) and AQP0 in the *p*12_1_ crystals formed with DMPG (AQP0_DMPG-p121_) (Fig. 3B). The models fit very well into the density maps, indicating that at a resolution of 7–8 Å the structure of AQP0 is the same in the AQP0_DMPC_, AQP0_DMPG-p4212_ and AQP0_DMPG-p121_ crystals.

Comparison of the pseudo-atomic models of the AQP0_DMPG-p121_ and AQP0_DMPG-p4212_ crystals with the AQP0_DMPC_ crystal reveals that the individual crystalline layers of the double-layered 2D crystals can be exactly superimposed on one another, demonstrating that the arrangement of the tetramers within each membrane is the same in all double-layered AQP0 2D crystals (S3 Fig.). While the arrangement of the tetramers within each membrane is unchanged, the interactions between the two membranes are unique for each of the three alternative crystal forms. In the AQP0_DMPC_ crystal form, tetramers in opposing membranes are exactly in register, creating the *p*422 symmetry (Fig. 3C). The projection map shows that this tetramer packing is conserved in AQP0 2D crystals grown with DMPG with *p*422 symmetry (Fig. 1C). In the AQP0_DMPG-p4212_ crystal form, a tetramer in one membrane is in the center of four tetramers in the opposing membrane, creating the *p*42_1_2 symmetry (Fig. 3A). This change in register corresponds to a shift of a tetramer in one membrane by half a unit cell length, 32.75 Å, with respect to the tetramer in the opposing membrane in both in-plane directions. In the AQP0_DMPG-p121_ form, a tetramer in one membrane is also shifted by 32.75 Å with respect to the tetramer in the opposing membrane in one in-plane direction but only by 28.7 Å in the other in-plane direction, thus creating the *p*12_1_ symmetry (Fig. 3B). In addition to the lateral translation of the two layers with respect to each other, the distances between the two layers in the *p*42_1_2 and *p*12_1_ crystal forms, measured as the distance between centroids representing the top and bottom tetramers, are 38.3 Å and 43.3 Å, respectively. Hence, the two layers in these crystal forms are closer together than in the *p*422 crystals grown with DMPC and EPL (47.0 Å) (Fig. 3, lower panels).

The changes in lateral and vertical positioning of the two membrane layers in AQP0_DMPG-p4212_, AQP0_DMPG-p121_, and AQP0_DMPC_ result in significantly different interactions between tetramers in the two layers. The tetramers in AQP0_DMPC_ are stacked directly on top of one another, and each tetramer thus forms junctional interactions with a single tetramer in the opposing layer (Fig. 3C) [4]. In contrast, each tetramer in AQP0_DMPG-p4212_ and AQP0_DMPG-p121_ contacts four tetramers in the opposing layer through interactions mediated mostly by single pairs of monomers (Fig. 3A, B). The interactions between the layers in AQP0_DMPG-p4212_ and AQP0_DMPG-p121_ must also be distinct, as the different registers between the two layers in these two crystal forms creates entirely different interacting surfaces (S2 Fig., middle panels).

### The formation of different AQP0 crystal forms depends on the DMPG concentration

**Fig 4:**
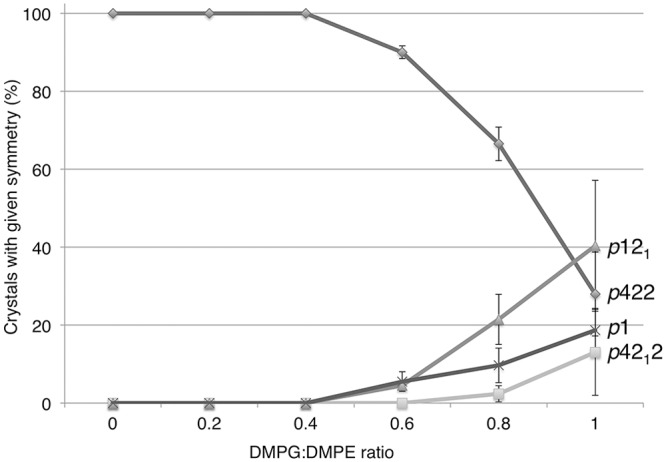
Percentage of AQP0 2D Crystals with a Given Symmetry Formed at Different DMPG:DMPE Ratios. Up to a DMPG concentration of 40% (w/w), all crystals showed *p*422 symmetry. At higher DMPG concentrations, the percentage of crystals with *p*422 symmetry decreased as increasingly more crystals with other symmetries formed. The results shown are averages of three independent experiments, and the bars represent standard deviation. See also S2 Table.

AQP0 was previously crystallized in the *p*422 plane group with EPL [6], which have PE as the major constituent, but also contain approximately 25% (w/w) PG lipids [18], suggesting that PG may influence crystal formation in a concentration-dependent manner. To investigate the role of PG concentration in defining the symmetry of AQP0 2D crystals, AQP0 was reconstituted with mixtures of DMPE, a lipid that forms exclusively AQP0 2D crystals with *p*422 symmetry (data not shown), and increasing amounts of DMPG (0%, 20%, 40%, 60%, 80%, and 100% (w/w)). These reconstitutions were repeated twice and approximately 30 crystals were imaged for each condition of the three independent reconstitutions. The symmetry of the imaged crystals was determined by using the program ALLSPACE [11]. The *p*422 crystal from was present in all conditions, but its abundance declined from 100% at 0%, 20% and 40% DMPG to a low of ~30% at 100% DMPG (Fig. 4, S2 Table). In the reconstitutions with higher DMPG concentrations, the other crystal forms replaced the *p*422 form, but their relative abundance varied between the three reconstitutions. Thus, DMPG concentrations of 60% or more destabilize the *p*422 packing but do not seem to favor one particular arrangement between the two layers.

### Model for DMPG-induced shifts between the two layers in AQP0 2D crystals

Our current data do not allow us to identify the molecular mechanisms by which DMPG induces shifts between the two crystalline layers in double-layered AQP0 2D crystals, but our results allow us to speculate on what may occur. AQP0 purified from the lens core is a mixture of full-length and truncated protein and runs on gel-filtration chromatography as two peaks that presumably represent a single AQP0 tetramer and two stacked tetramers, respectively [4]. We therefore speculate that upon reconstitution of AQP0 with DMPG, the crystals initially have *p*422 symmetry, essentially representing arrays of stacked tetramers, as those seen in crystals formed with DMPC and EPL. However, crystals with *p*422 symmetry appear not to be stable in the context of a DMPG lipid bilayer, and the two layers shift with respect to each other. As a result we observe multiple different crystal morphologies, including several with *p*1 symmetry, which represent essentially random shifts between the two layers. The two most common crystal forms, the *p*12_1_ and *p*42_1_2 crystals, seem to provide the strongest interactions between the two layers in the context of a DMPG lipid bilayer based on their relative abundance in AQP0 2D crystals formed with high concentrations of DMPG.

While our pseudo-atomic models of the AQP0 crystals with *p*42_1_2 and *p*12_1_ symmetry do not allow us to describe the interactions of lipids with AQP0, they do provide information on the packing of the tetramers in these crystal forms. In the DMPG crystals with *p*42_1_2 and *p*12_1_ symmetry, the shifts between the two layers are accompanied by a reduction in the distance between the two layers (Fig. 3). Just like AQP0, AQP4 also forms double-layered 2D crystals in which the register and distance between the two layers vary [17]. In the case of AQP4, PE lipids were necessary for crystal formation, and the PE head groups in one layer were found to interact with AQP4 in the opposing membrane [19]. Since the distance between the two layers in the AQP0 2D crystals with *p*42_1_2 and *p*12_1_ symmetry, 38.3 Å and 43.3 Å, respectively, is smaller than the distance between the two layers in the AQP4 2D crystals, 45 Å, it appears likely that the PG head groups in one layer of the AQP0 2D crystals could also make contacts with AQP0 tetramers in the opposing layer. Such interactions between the extracellular loops of AQP0 in one layer with the PG head groups of lipids in the other layer, made possible by the shorter distance between the two layers in crystals with *p*42_1_2 and *p*12_1_ symmetry, may lead to the stabilization of AQP0 2D crystals with these symmetries.

The question remains, however, why PG head groups would destabilize AQP0 crystals with *p*422 symmetry. The atomic model obtained with double-layered AQP0 2D crystals showed that junction interactions are mostly mediated by proline residues [4]. It may thus be that the uncharged glycerol head group of DMPG can compete with these interactions. Hence, although PG lipids are classified as anionic lipids, it would actually be the uncharged character of the glycerol moiety that would be responsible for the destabilization of the *p*422 symmetry. This model would also explain the gradual effect of DMPG, with increasing DMPG concentrations, i.e., increasing concentrations of glycerol moieties increasingly destabilizing the interaction between the extracellular loops of AQP0 tetramers in the opposing layers. To test this idea, we grew AQP0 2D crystals with other anionic phospholipids.

### Effect of other phospholipid head groups on AQP0 2D crystals

Like lipids with a PG head group, lipids with phosphatidylserine (PS) and phosphatidic acid (PA) head groups are classified as anionic lipids. However, due to their different size and charge distribution, the three head groups have very different chemical properties. PA is simplest, formed from just the primary phosphate group attached to the glycerol backbone. PG and PS both possess large moieties bound to the phosphodiester group, but in PG the moiety is an uncharged glycerol, while it is a zwitterionic serine in PS (S1A Fig.). Again, to isolate the specific effect of the head group, we used DMPS and DMPA for our experiments, which have the same two myristoyl chains as DMPC and DMPG.

**Fig 5:**
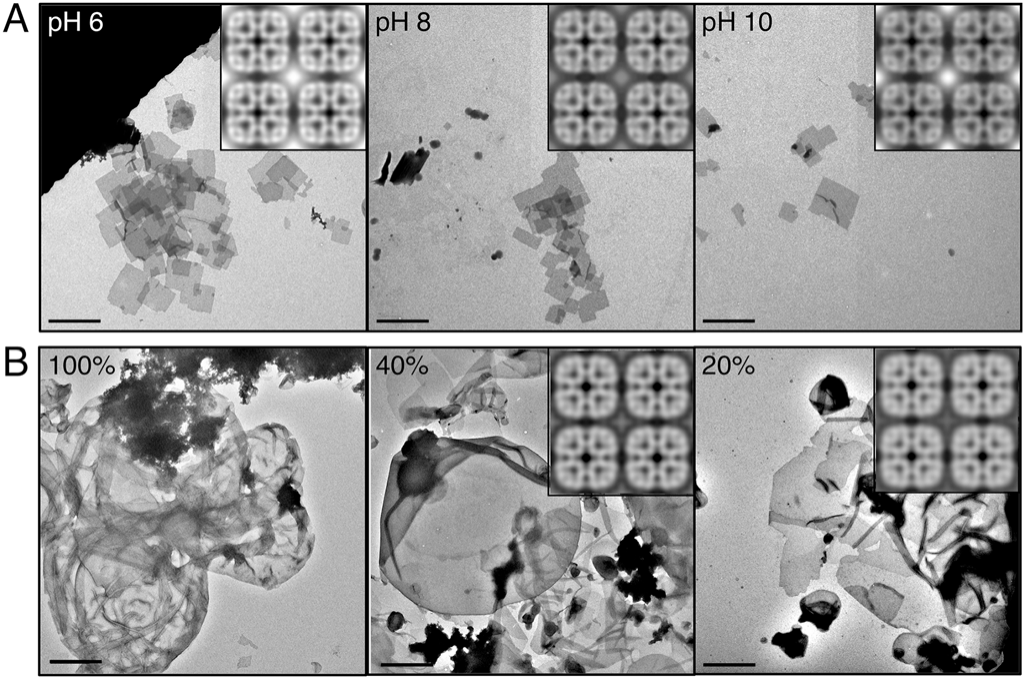
Representative Images of Negatively Stained Samples of AQP0 Reconstituted with the Anionic Lipids DMPS and DMPA. **A** AQP0 reconstituted with DMPS at pH 6.0 yielded 2D crystals that showed a pronounced tendency to stack (left panel). Incubation of these crystals with pH 8 buffer resulted in partial unstacking of the crystals (middle panel), and incubation with pH 10 buffer completely separated the crystals (right panel). All crystals under all pH conditions always showed *p*422 symmetry (inset in left panel). **B** Reconstitution of AQP0 with DMPA at pH 6.0 resulted in protein aggregates and presumably mostly empty vesicles (left panel). Only DMPE/DMPA mixtures that contained 20% or 40% DMPA (w/w) (middle and right panel, respectively) resulted in the formation of 2D crystals, which were always of *p*422 symmetry (insets). Scale bars are 2 μm.

Reconstitution of AQP0 with DMPS resulted in large and well-ordered double-layered 2D crystals that had a pronounced tendency to stack (Fig. 5A, left panel), allowing cryo-EM images of trehalose-embedded samples to be recorded only of the few crystalline areas that were not stacked. Processing of these crystalline areas showed that the crystals had *p*422 symmetry, as seen in the projection map at 5 Å resolution that was obtained by merging 16 images (Fig. 5A, left panel, inset, and S3 Table).

Stacking of AQP0 2D crystals was not seen with any lipid other than DMPS. The pKa of the carboxylic acid on the PS head group is approximately 5.5, very similar to the pH of our reconstitution buffer, indicating that under our reconstitution conditions, only a fraction of the molecules will be negatively charged [20]. We therefore tested whether changing the pH would reduce crystal stacking. Indeed, while diluting the crystal solution with a pH 4 buffer had no effect, diluting the crystal solution with buffers of pH 8 and 10 markedly reduced the stacking without altering crystal packing (Fig. 5A, middle and right panels), suggesting that deprotonation of the carboxylic acid of the PS head group in the crystalline layers eliminated the stacking interactions. Reconstitution of AQP0 with DMPS using dialysis buffers of pH 8 and 10 usually resulted in lipid-protein aggregates, indicating that reconstitution of AQP0 with DMPS is sensitive to pH, but in the one instance (out of 18 attempts) when 2D crystals formed, these were well separated and had *p*422 symmetry.

Under all of the tested conditions, AQP0 exclusively formed double-layered 2D crystals with *p*422 symmetry in the presence of DMPS and thus all anionic lipids do not destabilize the junction-forming proline—proline interactions as does DMPG. However, DMPS does promote interactions between different 2D crystals, which must occur through the crystal surfaces exposing the cytoplasmic surfaces of AQP0, thus causing stacking of multiple crystals. Stacking is prominent at pH 6, where the serine moiety of PS is mostly zwitterionic. At higher pH values, the amino group of the PS head group will become increasingly neutral, no longer compensating for the negative charge of the carboxyl group. Our results thus suggest that stacking interactions depend on a zwitterionic PS head group, while higher pH values prevent crystal stacking due to the uncompensated negative charge of the carboxyl group of the PS head group.

Lastly, we attempted to reconstitute AQP0 with DMPA to test the influence of the negatively charged phosphate group itself. Despite repeated attempts to reconstitute AQP0 with DMPA alone, no crystals were obtained. Instead the samples contained vesicles (presumably empty as power spectra of images of the vesicles prepared by negative staining showed no indication of powder diffraction) and large protein aggregates (Fig. 5B, left panel). 2D crystals could only be obtained when AQP0 was reconstituted with mixtures of DMPA and DMPE, and even then only at DMPA concentrations of 20% and 40% (w/w) (Fig. 5B, middle and right panels). At higher DMPA concentrations, the reconstitutions were indistinguishable from reconstitutions with 100% DMPA. Processing of images taken of trehalose-embedded AQP0 crystals that formed with 20% and 40% DMPA showed that all crystals had *p*422 symmetry, as illustrated by the resulting projection maps at 6.5 Å resolution (Fig. 5B, middle and right panels, insets, and S3 Table). In addition, the mixtures of DMPA and DMPE produced vesicular crystals, whereas all other anionic lipids yielded crystalline sheets. Since the reconstitution conditions were always the same and since the protein does not adopt different conformations, this finding indicates that the lipid head group is one of the factors that determines the crystal morphology.

The inability to reconstitute AQP0 with DMPA was unexpected, as we were able to reconstitute AQP0 with all other lipids and lipid mixtures we tested. One potential explanation for the incompatibility of PA with the formation of AQP0 2D crystals may be the small size of the head group, which has a cross-sectional area (24 Å^2^) that is smaller than that of the two acyl chains (39 Å^2^) [21], which can result in the formation of non-bilayer structures under certain conditions [22]. We do not detect any of these non-bilayer structures, but it may be possible that the unique shape and/or charge distribution of the phosphate head group may inhibit formation of mixed protein/lipid/detergent micelles, thereby excluding AQP0 from forming membranes.

http://avantilipids.com/index.php?option=com_content&view=article&id=1702&itemid=421

### Role of lipids in AQP0-mediated membrane junctions

We have presented several different types of AQP0 2D crystals that form in the presence of high concentrations of different anionic phospholipids. These crystals demonstrate that lipid head groups can influence the interactions between membrane proteins, but it is unlikely that they affect the physiological interactions that occur between AQP0 arrays in adjacent lens fiber cells. Lens fiber cell membranes contain high concentrations of sphingolipids and cholesterol, two neutral lipid species, no detectable levels of PG, and very small quantities of PS and PA [23]. In membranes formed from zwitterionic lipids, AQP0 always adopts the *p*422 crystal form, in which the junctional interactions are mediated by exposed proline residues on the extracellular loops. The *p*422 crystal form is therefore the likely arrangement of tetramers in native AQP0-mediated thin junctions between lens fiber cells. Furthermore, the lipid composition of the membrane is unlikely to be a physiological regulator of AQP0 function. During maturation, lens fiber cells extrude their organelles [24], making it highly unlikely that the lens fiber cell membranes can undergo dramatic changes in their lipid composition. However, the propensity of AQP0 to form high-quality 2D crystals with different lipids makes this protein interesting beyond its biological functions, because it provides the unique possibility to study the structural characteristics of generic lipid—protein interactions.

## Conclusions

In this study we show that the specific chemistry of lipid head groups have distinct effects on 2D crystals formed by AQP0. DMPG affects how the extracellular surfaces of the crystalline layers interact with each other, resulting in lateral and vertical shifts between the two layers, presumably due to the uncharged glycerol head group competing with the Pro—Pro interactions that normally stabilize the AQP0 junction-forming interactions. The PG head group has, however, no effect on the cytoplasmic surfaces of the 2D crystals. DMPS has the opposite effects. Its head group always has at least one charge, which presumably prevents it from interfering with the AQP0 junction-forming proline—proline interactions. At low pH, however, protonation of the carboxylic acid group induces interactions between different double-layered AQP0 2D crystals through their exposed cytoplasmic surfaces. Finally, high concentrations of DMPA appear to be incompatible with 2D crystallization and even mere reconstitution of AQP0, suggesting that head group moieties are essential for accommodating proteins in a lipid bilayer. Thus, while DMPG, DMPS and DMPA are all anionic phospholipids, each has a unique effect on 2D crystallization of AQP0. These results demonstrate that the specific chemical interactions allowed by different head groups are as important as their overall charge. The lipid head groups create distinct surface properties of the membrane that also modify the properties of the embedded proteins. High-resolution structures of AQP0 with anionic lipids will be needed to elucidate the molecular details of the different interactions these lipids form with AQP0.

## Supporting Information

S1 Fig Chemical Structures of Anionic Phospholipids and Electron Diffraction of an AQP0 2D Crystal Grown With DMPG. **A** The lipids used to grow 2D crystals of AQP0 are dimyristoyl phosphatidylglycerol (DMPG), dimyristoyl phosphatidylserine (DMPS), and dimyristoyl phosphatidic acid (DMPA). The shaded areas indicate the head groups of the lipids, which consist of the phosphate group (darker shading) and in the case of PG and PS an additional head group moiety (lighter shading). The red and blue circles indicate negatively and positively charged groups, respectively. **B** Representative electron diffraction pattern of an untilted AQP0 2D crystal formed with DMPG. After background subtraction, diffraction spots are visible beyond 2 Å resolution. Reflection (22, 27) is circled in black and corresponds to a resolution of 1.9 Å. Scale bar is (10 Å)^–1^. **C** Enlarged view of the area indicated by the dashed square in (B). Scale bar is (10 Å)^–1^.
(TIF)

S2 Fig Density Maps of AQP 2D Crystals Grown with DMPG. **A** Density map of AQP0 2D crystal with *p*42_1_2 at 7 Å resolution. **B** Density map of AQP0 2D crystal with *p*12_1_ symmetry before non-crystallographic symmetry averaging at 8 Å resolution.
(TIF)

S3 Fig Superimposition of the Two Layers in Different AQP0 2D Crystals. Superimposition of the two pseudo-atomic models of the top layers (A) and the bottom layers (B) of the AQP0_DMPG-p121_ (green) and AQP0 _DMPG-p4212_ (orange) crystals with the AQP0_DMPC_ crystal (blue) shows that the arrangement of the tetramers in the two layers is the same in all double-layered AQP0 2D crystals.
(TIF)

S1 Table Internal phase residuals of all possible rectangular two-sided plane groups for the images shown in Fig. 1C. Internal phase residuals were determined using the program ALLSPACE [11] using spots from IQ1 to IQ5 to a resolution of 6 Å. Only plane groups compatible with the AQP0 lattice are shown.
(DOCX)

S2 Table The percentages of different crystal forms of AQP0 with the lipid mixtures of DMPE and DMPG. (DOCX)

S3 Table Image analysis of AQP0 2D crystals grown with DMPS (AQP0_DMPS_) and with DMPA/DMPE mixtures (AQP0_DMPA/DMPE_). (DOCX)
