# Supplementary figures and images for: Effect of Lipid Head Groups on Double-Layered Two-Dimensional Crystals Formed by Aquaporin-0
Source: PLoS One. 2015 Jan 30;10(1):e0117371. doi: 10.1371/journal.pone.0117371 (PMC4311914; doi:10.1371/journal.pone.0117371)

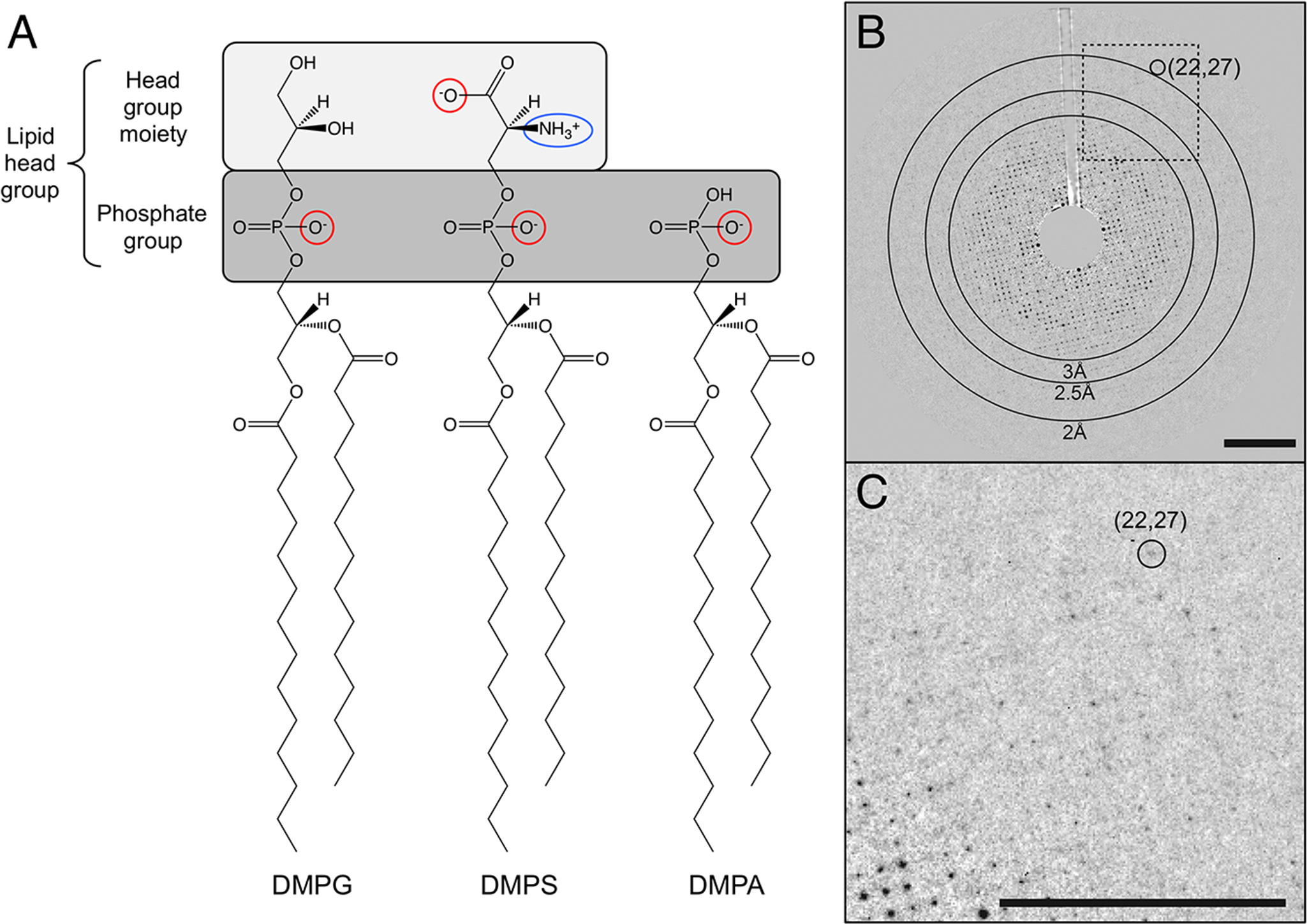

Supplement: S1 Fig — A The lipids used to grow 2D crystals of AQP0 are dimyristoyl phosphatidylglycerol (DMPG), dimyristoyl phosphatidylserine (DMPS), and dimyristoyl phosphatidic acid (DMPA). The shaded areas indicate the head groups of the lipids, which consist of the phosphate group (darker shading) and in the case of PG and PS an additional head group moiety (lighter shading). The red and blue circles indicate negatively and positively charged groups, respectively. B Representative electron diffraction pattern of an untilted AQP0 2D crystal formed with DMPG. After background subtraction, diffraction spots are visible beyond 2 Å resolution. Reflection (22, 27) is circled in black and corresponds to a resolution of 1.9 Å. Scale bar is (10 Å)–1. C Enlarged view of the area indicated by the dashed square in (B). Scale bar is (10 Å)–1. (TIF) [file pone.0117371.s001.tif]

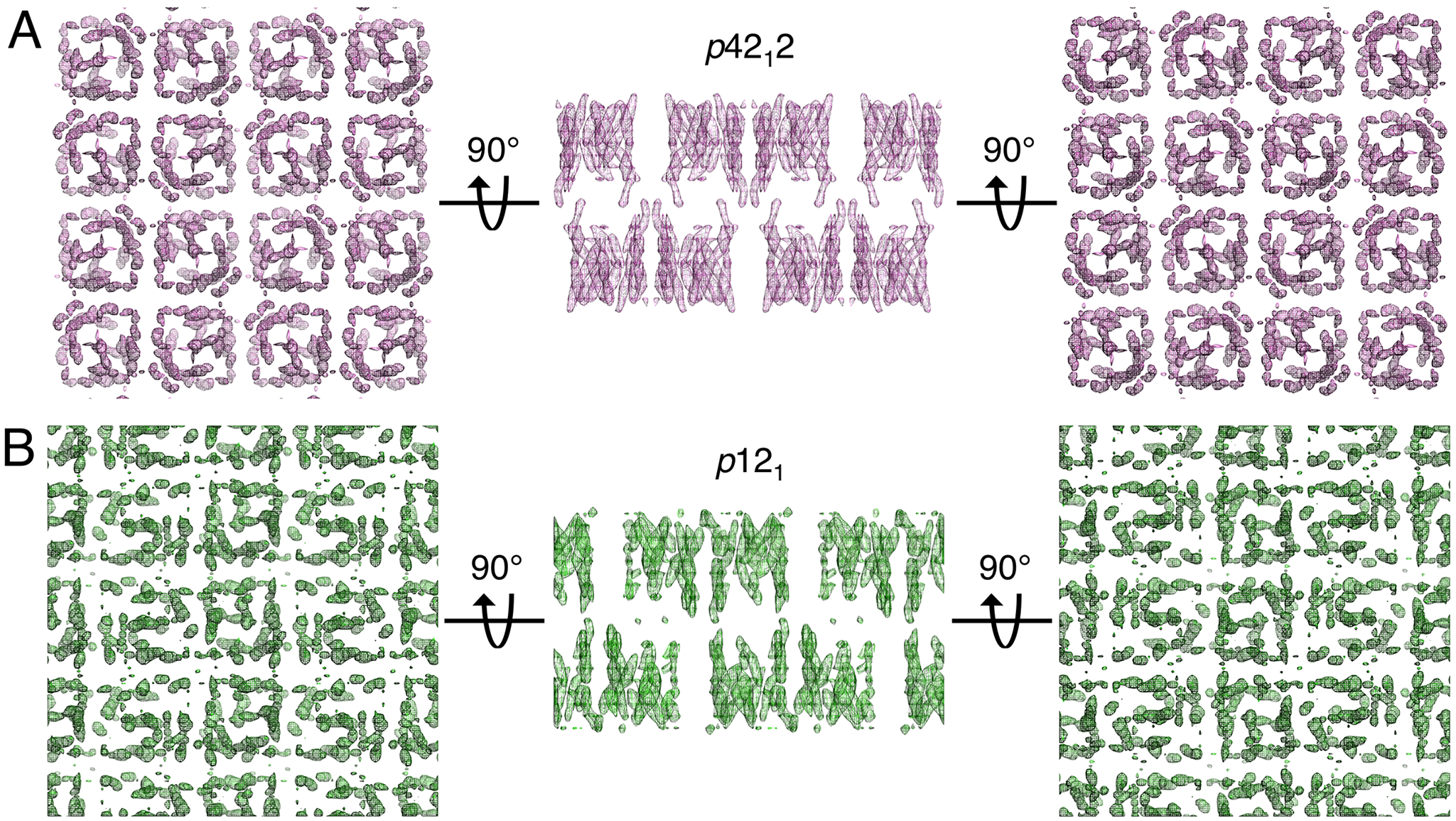

Supplement: S2 Fig — A Density map of AQP0 2D crystal with p4212 at 7 Å resolution. B Density map of AQP0 2D crystal with p121 symmetry before non-crystallographic symmetry averaging at 8 Å resolution. (TIF) [file pone.0117371.s002.tif]

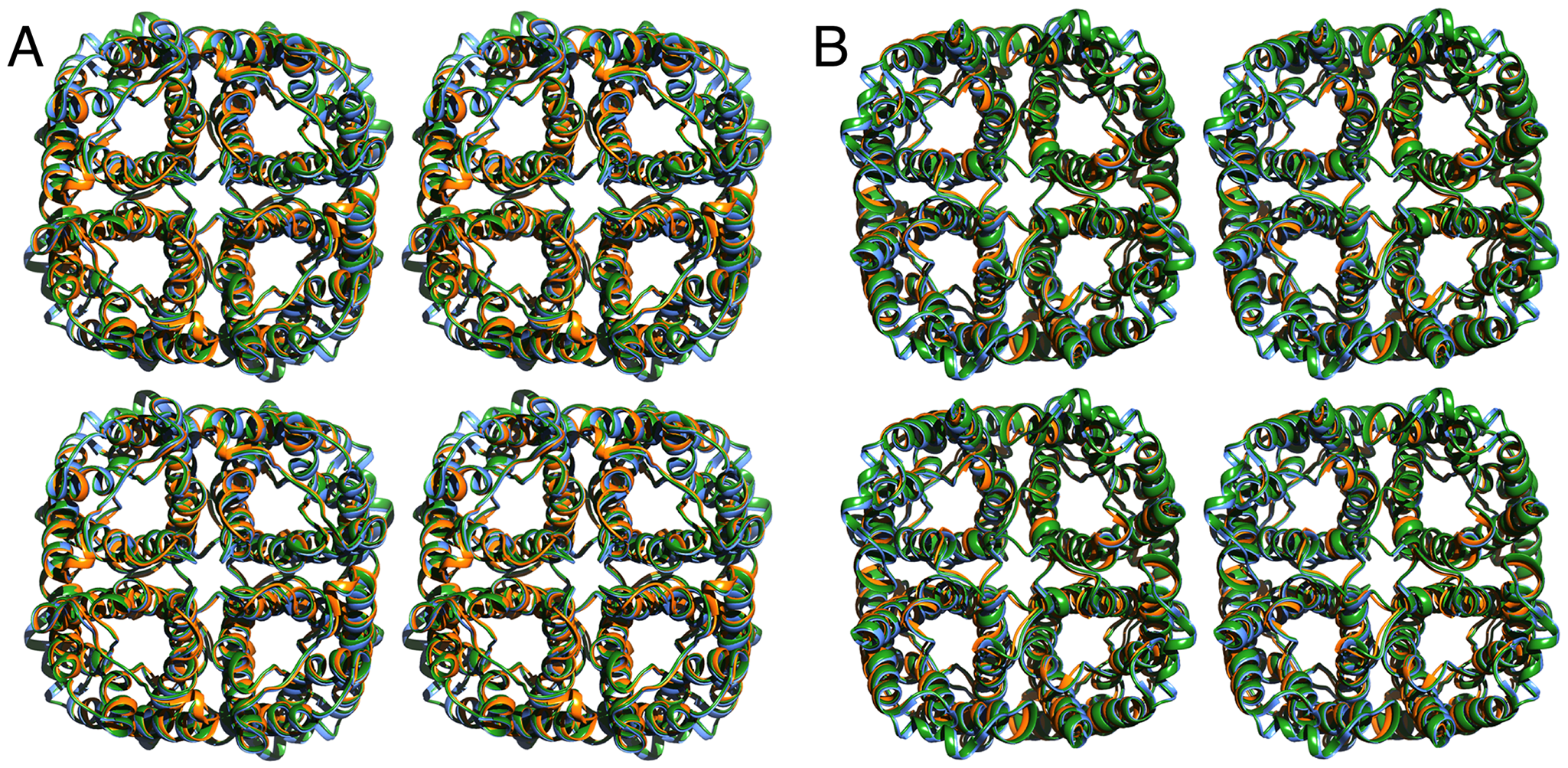

Supplement: S3 Fig — Superimposition of the two pseudo-atomic models of the top layers (A) and the bottom layers (B) of the AQP0DMPG-p121 (green) and AQP0 DMPG-p4212 (orange) crystals with the AQP0DMPC crystal (blue) shows that the arrangement of the tetramers in the two layers is the same in all double-layered AQP0 2D crystals. (TIF) [file pone.0117371.s003.tif]
