# Supplementary material for: Effect of Lipid Head Groups on Double-Layered Two-Dimensional Crystals Formed by Aquaporin-0
Source: PLoS One. 2015 Jan 30;10(1):e0117371. doi: 10.1371/journal.pone.0117371 (PMC4311914; doi:10.1371/journal.pone.0117371)
Supplement: S1 Table — Internal phase residuals were determined using the program ALLSPACE [11] using spots from IQ1 to IQ5 to a resolution of 6 Å. Only plane groups compatible with the AQP0 lattice are shown. (DOCX) [file pone.0117371.s004.docx]

**Table S1. Internal phase residuals of all possible rectangular two-sided plane groups for the images shown in Figure 1C.**

|  | **Two-sided plane group** | **Phase residual (degrees)^a^** | **Number of comparisons** | **Target residual (degrees)^b^** |
| --- | --- | --- | --- | --- |
| ***p*422 symmetry** | *p*1 | 19.4^c^ | 378 |  |
|  | *p*2 | 35.3^d^ | 189 | 27.9 |
|  | *p*12b | 15.6^f^ | 156 | 19.9 |
|  | *p*12_1_b | 82.6 | 156 | 19.9 |
|  | *c*12b | 15.6^f^ | 156 | 19.9 |
|  | *p*222 | 27.5^e^ | 502 | 22.6 |
|  | *p*222_1_b | 61.8 | 502 | 22.6 |
|  | *p*22_1_2_1_ | 68.2 | 502 | 22.6 |
|  | *c*222 | 27.5^e^ | 502 | 22.6 |
|  | *p*4 | 26.5^e^ | 533 | 22.4 |
|  | ***p*422** | **24.6^e^** | **1170** | **20.8** |
|  | *p*42_1_2 | 60.4 | 1170 | 20.8 |
|  | | | | |
| ***p*42_1_2 symmetry** | *p*1 | 22.2^c^ | 198 |  |
|  | *p*2 | 41.9^d^ | 99 | 32.2 |
|  | *p*12b | 77.3 | 78 | 23.0 |
|  | *p*12_1_b | 22.5^f^ | 78 | 23.0 |
|  | *c*12b | 77.3 | 78 | 23.0 |
|  | *p*222 | 67.0 | 256 | 26.1 |
|  | *p*222_1_b | 58.3 | 256 | 26.1 |
|  | *p*22_1_2_1_ | 30.1^e^ | 256 | 26.1 |
|  | *c*222 | 67.0 | 256 | 26.1 |
|  | *p*4 | 31.5^d^ | 267 | 25.9 |
|  | *p*422 | 57.8 | 592 | 23.9 |
|  | ***p*42_1_2** | **27.6^e^** | **592** | **23.9** |
|  | | | | |
| ***p*12_1_ symmetry** | *p*1 | 17.1^c^ | 238 |  |
|  | *p*2 | 60.3 | 119 | 17.7 |
|  | *p*12b | 81.8 | 108 | 17.7 |
|  | ***p*12_1_b** | **15.2^e^** | **108** | **17.7** |
|  | *c*12b | 81.8 | 108 | 17.7 |
|  | *p*222 | 73.2 | 333 | 19.8 |
|  | *p*222_1_b | 83.1 | 333 | 19.8 |
|  | *p*22_1_2_1_ | 59.6 | 333 | 19.8 |
|  | *c*222 | 73.2 | 333 | 19.8 |
|  | *p*4 | 57.3 | 339 | 19.8 |
|  | *p*422 | 67.8 | 771 | 18.3 |
|  | *p*42_1_2 | 67.5 | 771 | 18.3 |
|  | | | | |
| ***p*1 symmetry** | ***p*1** | **18.8^c^** | **110** |  |
|  | *p*2 | 55.3 | 55 | 27.1 |
|  | *p*12b | 62.2 | 45 | 19.7 |
|  | *p*12_1_b | 78.7 | 45 | 19.7 |
|  | *c*12b | 62.2 | 45 | 19.7 |
|  | *p*222 | 65.3 | 145 | 21.9 |
|  | *p*222_1_b | 68.4 | 145 | 21.9 |
|  | *p*22_1_2_1_ | 73.5 | 145 | 21.9 |
|  | *c*222 | 65.3 | 145 | 21.9 |
|  | *p*4 | 60.5 | 151 | 21.8 |
|  | *p*422 | 62.9 | 336 | 20.1 |
|  | *p*42_1_2 | 72.7 | 336 | 20.1 |

Internal phase residuals were determined using the program ALLSPACE (Valpuesta et al., 1994) using spots from IQ1 to IQ5 to a resolution of 6 Å. Only plane groups compatible with the AQP0 lattice are shown.

^a^Phase residual *versus* other spots (90° random).

^b^Target residual based on the statistics taking Friedel weight into account.

^c^Note that no phase comparison is possible in space group *p*1, so the listed numbers are theoretical phase residuals based on the signal-to-noise ratio of the observed diffraction spots in the Fourier transform.

^d^Within 10° of target residual.

^e^Within 5° of target residual.

^f^Symmetry that yields a residual better than the target residual.
