# Supplementary material for: Effect of Lipid Head Groups on Double-Layered Two-Dimensional Crystals Formed by Aquaporin-0
Source: PLoS One. 2015 Jan 30;10(1):e0117371. doi: 10.1371/journal.pone.0117371 (PMC4311914; doi:10.1371/journal.pone.0117371)
Supplement: S2 Table — (DOCX) [file pone.0117371.s005.docx]

**Table S2. The percentages of different crystal forms of AQP0 with the lipid mixtures of DMPE and DMPG.**

|  |  | **DMPG concentration (w/w)** | | | | | |
| --- | --- | --- | --- | --- | --- | --- | --- |
|  |  | 0% | 20% | 40% | 60% | 80% | 100% |
|  |  | Number of images / Percentage of crystal population | | | | | |
| **Repeat 1** | *p*422 | 22 / 100% | 30 / 100% | 27 / 100% | 44 / 89.8% | 18 / 66.7% | 11 / 39.3% |
|  | *p*42_1_2 | 0 / 0% | 0 / 0% | 0 / 0% | 0 / 0% | 1 / 3.70% | 7 / 25.0% |
|  | *p*12_1_ | 0 / 0% | 0 / 0% | 0 / 0% | 2 / 4.08% | 4 / 14.8% | 6 / 21.4% |
|  | *p*1 | 0 / 0% | 0 / 0% | 0 / 0% | 3 / 6.12% | 4 / 14.8% | 4 / 14.3% |
| **Repeat 2** | *p*422 | 24 / 100% | 25 / 100% | 34 / 100% | 33 / 91.7% | 18 / 62.1% | 5 / 17.9% |
|  | *p*42_1_2 | 0 / 0% | 0 / 0% | 0 / 0% | 0 / 0% | 1 / 3.40% | 3 / 10.7% |
|  | *p*12_1_ | 0 / 0% | 0 / 0% | 0 / 0% | 2 / 5.56% | 8 / 27.6% | 13 / 46.4% |
|  | *p*1 | 0 / 0% | 0 / 0% | 0 / 0% | 1 / 2.78% | 2 / 6.90% | 7 / 25.0% |
| **Repeat 3** | *p*422 | 23 / 100% | 23 / 100% | 24 / 100% | 23 / 88.5% | 29 / 70.7% | 8 / 26.7% |
|  | *p*42_1_2 | 0 / 0% | 0 / 0% | 0 / 0% | 0 / 0% | 0 / 0% | 1 / 3.33% |
|  | *p*12_1_ | 0 / 0% | 0 / 0% | 0 / 0% | 1 / 3.85% | 9 / 22.0% | 16 / 53.3% |
|  | *p*1 | 0 / 0% | 0 / 0% | 0 / 0% | 2 / 7.69% | 3 / 7.32% | 5 / 16.7% |
| **Average** | *p*422 | 100% | 100% | 100% | 90.0% | 66.5% | 28.0% |
|  | *p*42_1_2 | 0% | 0% | 0% | 0% | 2.33% | 12.9% |
|  | *p*12_1_ | 0% | 0% | 0% | 4.47% | 21.5% | 40.4% |
|  | *p*1 | 0% | 0% | 0% | 5.53% | 9.67% | 18.7% |
| **STD** | *p*422 | 0% | 0% | 0% | 1.61% | 4.30% | 10.8% |
|  | *p*42_1_2 | 0% | 0% | 0% | 0% | 2.06% | 11.0% |
|  | *p*12_1_ | 0% | 0% | 0% | 0.93% | 6.42% | 16.8% |
|  | *p*1 | 0% | 0% | 0% | 2.51% | 4.44% | 5.61% |
