# Supplementary material for: Effect of Lipid Head Groups on Double-Layered Two-Dimensional Crystals Formed by Aquaporin-0
Source: PLoS One. 2015 Jan 30;10(1):e0117371. doi: 10.1371/journal.pone.0117371 (PMC4311914; doi:10.1371/journal.pone.0117371)
Supplement: S3 Table — (DOCX) [file pone.0117371.s006.docx]

**Table S3. Image analysis of AQP0 2D crystals grown with DMPS (AQP0_DMPS_) and with DMPA/DMPE mixtures (AQP0_DMPA/DMPE_).**

|  | AQP0_DMPS_ | | | AQP0_DMPA/DMPE_ | | |
| --- | --- | --- | --- | --- | --- | --- |
| pH | 6.0 | 8.0 | 10.0 |  | |  |
| DMPA content (w/w) |  |  |  | 20% | | 40% |
| **Data analysis** | | | | | | |
| Image collected | 16 | 34 | 40 | 34 | | 30 |
| Cell dimensions | *a* = 65.5 Å, *b* = 65.5 Å, and γ = ° | | | | | |
| Space group (from ALLSPACE) | *p*422 | | | | | |
|  | | | | | | |
| **Data merging** | | | | | | |
| Number of images merged | 10 | 10 | 10 | | 10 | 10 |
| Number of the spots | 2,179 | 2,375 | 2,306 | | 1,362 | 1,468 |
| Resolution limit for merging | 5.0 Å | 5.0 Å | 5.0 Å | | 6.5 Å | 6.5 Å |
| Phase residuals |  | | | | | |
| 1,000 Å – 5.0 Å (overall) | 32.7º | 32.7º | 31.0º | |  |  |
| 6.0 Å – 5.6 Å | 29.9º | 28.9º | 24.3º | |  |  |
| 5.6 Å – 5.3 Å | 36.6º | 34.7º | 33.1º | |  |  |
| 5.3 Å – 5.0 Å | 35.8º | 34.4º | 33.4º | |  |  |
|  | | | | | | |
| 1,000 Å – 6.5 Å (overall) |  |  |  | | 27.5º | 32.3º |
| 7.8 Å – 7.3 Å |  |  |  | | 20.7º | 26.6º |
| 7.3 Å – 6.9 Å |  |  |  | | 22.1º | 35.3º |
| 6.9 Å – 6.5 Å |  |  |  | | 38.2º | 40.9º |
